# ASSESSMENT OF INTEROBSERVER RELIABILITY FOR THE LETOURNEL AND JUDET CLASSIFICATION

**DOI:** 10.1590/1413-785220243201e267640

**Published:** 2024-03-22

**Authors:** Mehmet Yucens, Ahmet Nadir Aydemir, Ahmet Fahir Demirkan

**Affiliations:** 1 Pamukkale University, Faculty of Medicine, Departament of Orthopaedics, Denizli, Turkey.

**Keywords:** Acetabulum, Orthopedic Surgeons, Pelvis, Fractures, Bone, Acetábulo, Cirurgiões Ortopédicos, Pelve, Fracturas Óseas

## Abstract

**Introduction:**

The Judet and Letournel classification is the most widely used classification system for acetabular fractures. Some complex fractures couldn't be classified according to this classification. The main purpose of this study was to evaluate the reliability of the Letournel and Judet classification system for acetabular fractures.

**Material and methods:**

10 acetabular fractures were analyzed among 17 orthopedic surgeons. The surgeons were asked to classify the fractures according to the Judet and Letournel classification. Their experience, the number of surgeries, and the incision type that the surgeon uses for the anterior part of the acetabulum were recorded.

**Results:**

The overall interobserver agreement for the Letournel classification was found to be poor, with a Kappa value of 0.287. The Kappa value for interobserver agreement was 0.224 for plain radiographs, 0.293 for 2D-CT, and 0.321 for 3D-CT scans. There was no significant difference between the incision types used by the surgeons. The highest reliability was determined among the surgeons who operate on 10-20 acetabular fractures per year, with a Kappa value of 0.309.

**Conclusion:**

This results revealed that the Judet and Letournel Judet classification is not sufficient to classify acetabular fractures because of unclassified fractures and the complex algorithm of the system. ***Level of Evidence III; Comparative Retrospective Study***.

## RESUMO

**Introdução:**

A classificação de Judet e Letournel é o sistema de classificação mais amplamente utilizado para fraturas acetabulares. Algumas fraturas complexas, porém, não puderam ser classificadas de acordo com esta classificação. O principal objetivo deste estudo foi avaliar a confiabilidade do sistema de classificação de Judet e Letournel para fraturas acetabulares.

**Material e métodos:**

Foram selecionadas aleatoriamente 10 fraturas acetabulares de um banco de dados. Participaram do estudo 17 cirurgiões ortopédicos. Foi solicitado aos cirurgiões que classificassem as fraturas de acordo com a classificação de Judet e Letournel. Suas experiências, o número de cirurgias e o tipo de incisão que o cirurgião utiliza para a parte anterior do acetábulo foram registrados.

**Resultados:**

A concordância interobservadores geral para a classificação de Judet e Letournel foi considerada fraca, com um valor de Kappa de 0,287. O valor de Kappa para a concordância interobservadores foi de 0,224 para radiografias simples, 0,293 para tomografias computadorizadas em 2D e 0,321 para tomografias computadorizadas em 3D. Não houve diferença significativa entre os tipos de incisão utilizados pelos cirurgiões. A maior confiabilidade foi determinada entre os cirurgiões que operam de 10 a 20 fraturas acetabulares por ano, com um valor de Kappa de 0,309.

**Conclusão:**

Os resultados revelaram que a classificação de Judet e Letournel não é suficiente para classificar fraturas acetabulares devido a fraturas não classificadas e ao algoritmo complexo do sistema. ***Nível de Evidência III; Estudo Comparativo Retorpectivo***.

## INTRODUCTION

The treatment of acetabular fractures is one of the most complicated situations in orthopaedic surgery. There has been a progressive increase in the number of cases, resulting from high-energy accidents and due to the improvement of emergency rescue systems, which are able to save the life of a polytrauma patient.^1^ Accurate classification of acetabular fractures is very important when selecting the correct surgical approach to enable the most effective surgical treatment.^2^ The need for an accurate and precise classification system has been long established as a cornerstone in modern fracture treatment.^3^ Judet and Letournel, whose treatise analyses fractures of the acetabulum, named the columns in reference to their double embryological origin. The iliopubic column (anterior column) extends from the superior iliac crest to the pubic symphysis. The thicker structure of the ilioischial column (posterior column) extends from the inferior sacroiliac joint and sciatic notch to the ischial tuberosity. Letournel and Judet developed a classification system which divides fracture types into one of five elementary (simple) and five associated (complex) patterns based on the column system. The Letournel and Judet classification requires 3 plain radiographs of the pelvis: an AP view, an obturator oblique view, and an iliac oblique view. The evaluation of acetabular fractures is difficult and the classification systems of Letournel^4,5^ and AO/OTA^6^ are complex. There is a current trend towards increased utilization of computed tomography (CT) imaging both in the general population and specifically in the emergency care setting.^7^ The integration of CT scans into common medical care of trauma patients has increased our ability to detect fractures of the quadrilateral surface, sacrum, acetabular roof, and posterior acetabular wall, to identify loose bodies in the hip, and provides a more complete understanding of acetabular fracture characteristics.

The reliability of the Letournel and Judet classification was investigated previous studies. Beaule et al. stuied the reliability wtihin the different experience levels of surgeons but the fracture types did not selected randomly.^8^ Hutt et al. classified the fractures according to the Letournel and Judet classification. They used a hundred radiograps three observers and they put a tab as unclassified.^9^

The main purpose of this study was to evaluate additional information for the reliability of the Letournel and Judet classification system with randomly selected radiograms offered to observers who had variable degrees clinically. Each observer was familiar with the Letournel classification. Evaluations and comparisons were made in respect of Kappa values for interobserver reliability, according to the type of incision used for anterior part fractures, the experience of the surgeons, using plain radiographs and the additional effect of two-dimensional CT (2D-CT) and three-dimensional CT (3D-CT) scans and the complexity of the fracture type.

## MATERIAL and METHODS

**Figure 1:**
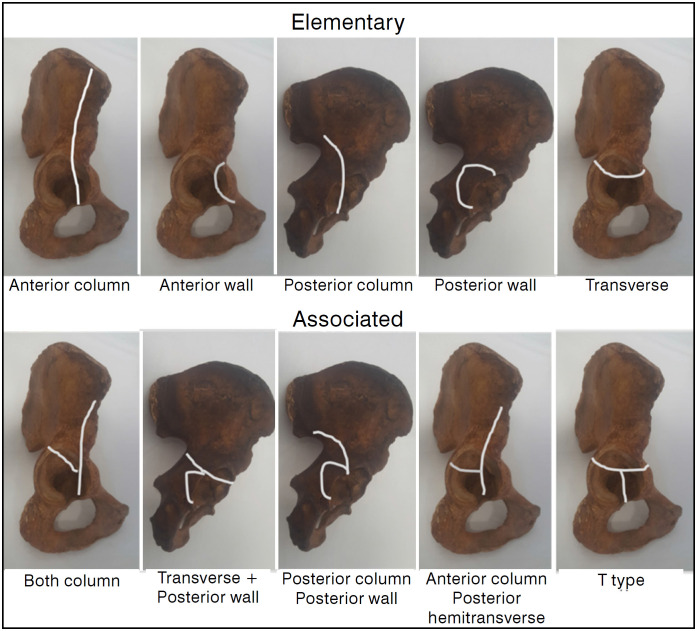
Elementary and associated types of acetabular fractures according to the Letournel classification.

In this study the reliability of the Letournel and Judet classification was investigated according to the participants' experience, the number of acetabular fracture surgeries performed per year, the incision type that the surgeon uses for the anterior part of the acetabulum and the effect of 2D and 3D CT scans. The Judet and Letournel classification divides acetabular fractures into 2 groups: elementary (simple) and associated (complex). The elementary fracture group includes anterior and posterior wall fractures, anterior and posterior column fractures, and transverse fractures. The associated acetabular fracture group includes both column fractures, anterior column posterior hemitransverse fractures, T-type fractures, and transverse or posterior column with posterior wall fractures. (Figure 1)

A total of 10 patients, aged >18 years, with 10 acetabular fractures were randomly selected from the hospital database. For each patient there was a complete set of radiographs, CT scans and 3D-CT scans taken within the last 3 years. The CT scans and the 3D-CT scans were uploaded to the internet in video format. A total of 17 orthopedic surgeons, all of whom were familiar with the Letournel and Judet classification and who were variable degrees in treating acetabular fractures, participated in the study. Standard forms were created and sent to the personal e-mail addresses of the participating observers. The participants were not given any clinical information regarding the demographic data, treatment methods or results of the patients. A schema and written explanation of the Letournel and Judet classification were given in the introduction of the Form (Figure 1). Each item in the survey had 10 different response options, comprising the types of acetabulum fracture according to the Letournel and Judet classification. The observers were asked to mark only one option in response to each item. The first 3 items were related to the experience of the surgeon, the number of acetabular fracture surgeries performed and the incision type that the surgeon uses for the anterior part of the acetabulum. The next item included three plain radiographs of the first patient and the participants were asked to classify the type of acetabulum fracture according to the Letournel and Judet classification. In the next item, the CT scans of the same patient were presented and the participants were again asked the type of acetabulum fracture according to the Letournel and Judet classification. The following item presented the 3D-CT scans of the same patient and the respondents were asked if there was any change to the diagnosis and if so, which fracture type did they now consider it to be. Ten patients radiographs, CT scans and 3D-CT scans of 10 patients were presented in this way. All the answers were collected and analysed statistically. Fleiss Kappa analysis was applied to analyse agreement between the surgeons. Statistical analysis was performed using R (version 3.4.3, Vienna, Austria) in RStudio software (Version 1.1.463 – © 2009-2018 RStudio, Inc.). The package used for the analysis was "irr". According to Landis and Koch, a Kappa value of 0.00–0.20 indicates slight agreement, 0.21–0.40 fair agreement, 0.41–0.60 moderate agreement, and Kappa 0.81 is considered to be almost perfect. ^10^

## RESULTS

**Figure 2:**
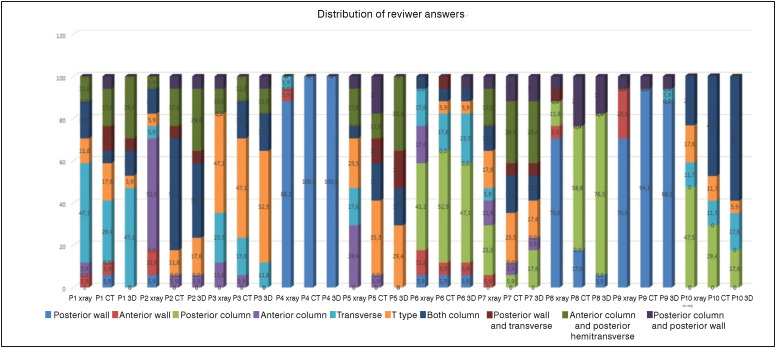
Distribution of observers answers.

In this study, 17 orthopaedic surgeons who had variable degrees clinically classified acetabular fractures according to the Letournel and Judet classification. The overall interobserver agreement for the Letournel classification was found to be poor with a Kappa value of 0.287 [Kappa (95 % CI), p<0.001]. When evaluating the interobserver agreement according to the selected incision, for ilioinguinal incision the Kappa value was found to be 0.282 [Kappa (95 % CI), p<0.001] and for modified medial Stoppa 0.281 [Kappa (95% CI), p<0.001]. The interobserver agreement according to the years of experience of the physician, was Kappa 0.262 for experience of 1-5 years [Kappa (95% CI), p<0.001], 0.303 for 5-10 years [Kappa (95% CI), p<0.001] and 0.238 for 10-20 years [Kappa (95% CI), p<0.001]. The interobserver agreement according to the physicians practice that the number of operated acetabular fracture per year, for 5-10 the kappa value was found 0.262 [Kappa (95% CI), p<0.001] for 10-20 0.309 [Kappa (95 % CI), p<0.001] and for 20 and over 0.278 [Kappa (95 % CI), p<0.001] (Table 1). The interobserver agreement for plain radiographs was Kappa value 0.224 for plain radiographs, 0.293 for 2D-CT, and 0.321 for 3D-CT. After the answers were collected the senior author classified the fracture types according to the Letournel classification (Table 2). The senior author's diagnostics were two posterior wall, two posterior column, one transverse, two t type, two anterior column posterior hemitransverse and one both column. Agreement percentage was 90% for posterior wall fractures, 68% for posterior column fractures. One t type fracture the agreement percentage was 49%. Another t type fracture the agreement percentage was 17%. (Table 2, Figure 2).

**Table 1:** The results of incision type, years of experience of the surgeon, number of operations performed per year, and the Kappa values.

	n	Kappa
Ilioinguinal	7	0.282
Modified medial Stoppa	10	0.281
1-5 years	7	0.262
5-10 years	7	0.303
10-20 years	3	0.238
5-10 operations per year	7	0.262
10-20 operations per year	6	0.309
20 + operations per year	4	0.278

**Table 2:** Senior author's diagnosis and percentages of the most given answers to the patients X-ray, 2-D CT, 3-D CT sans.

Patient	Senior author's diagnosis	X ray; most given answer/percentage		2-D CT; most given answer/percentage		3-D CT most given answer/percentage	
1	Transverse	Transverse	47.1	Transverse	29.4	Transverse	47.1
2	T type	Anterior column	52.9	Both column	52.9	Both column	35.3
3	T type	T type	47.1	T type	47.1	T type	52.9
4	Posterior wall	Posterior wall	88.2	Posterior wall	100	Posterior wall	100
5	Anterior column posterior hemitransverse	Anterior column	29.4	T type	35.3	Anterior column posterior hemitransverse	35.3
6	Posterior column	Posterior column	41.2	Posterior column	52.9	Posterior column	47.1
7	Anterior column posterior hemitransverse	Posterior column	23.5	Anterior column posterior hemitransverse	29.4	Anterior column posterior hemitransverse	29.4
8	Posterior column	Posterior wall	70.6	Posterior column	58.8	Posterior column	76.5
9	Posterior wall	Posterior wall	70.6	Posterior wall	94.1	Posterior wall	88.2
10	Both column	Posterior column	47.5	Both column	47.5	Both column	59

## DISCUSSION

**Figure 3:**
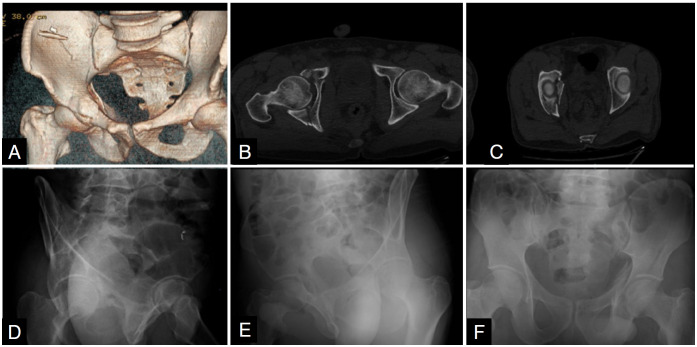
Patient eight radiograms; A: 3D-CT scan; B,C: 2D-CT scan; D: obturator oblique xray; E: iliac oblique xray; F: AP pelvis xray.

In this study the overall Kappa for the Letournel and Judet classification was found to be poor at 0.287. The study results also showed that the incision type preferred by the surgeon for anterior part fractures of the acetabulum did not change the reliability of the Letournel and Judet classification. In 2D CT, and 3D CT scans the kappa vale were increased and more importantly the agreement percentages were İncreased (Table 2, Figure 2). The experience of the surgeon partially increased the reliability but not to a significant degree. In the study by Beaulé et al., the senior author had originally classified the fractures at the time of surgery and patient radiographs were selected to include every type of fracture.^8^ The study was conducted with three groups of three orthopaedic surgeons: group 1, surgeons who had studied under Letournel, group 2, surgeons who specialized in acetabular fracture surgery, and group 3, general trauma surgeons. The interobserver reliability without and with CT during the first session was 0.70 and 0.74, respectively; 0.71 for group 1, 0.69 for group 2, and 0.51 for group 3. In the current study, radiographs and CT scans of randomly selected patients were used and after the observers results were collected, the senior author classified the fractures. The Kappa value was determined for overall, plain radiographs, 2D CT, and 3D CT scans and the questions were studied separately. In this study the kappa values was lower than Beaule study but the percent agreement was similar among elementary fracture types; posterior wall fractures was % 92 in Beaule, in this study was was % 90. For the associated fracture types they found percent agreement for t type 49 %, in this study percent agreement for one t type fracture which could be classify with Letournel was 49 % but another t type fracture percent agreement was % 17, the observers classified that as both column 35 % (Figure 3). In a study by Hutt et. al, 4 surgeons experienced in acetabular fracture surgery classified acetabulum fractures according to the Letournel and Judet classification and if they thought a fracture could not be classified, it was noted as unclassifiable. In that study, the overall Kappa value was 0.43 for plain radiographs and 0.54 for CT. When the unclassifiable fracture patterns were removed, inter-observer agreement was substantially improved to κ = 0.65 for radiographs alone, and near perfect κ = 0.80, with the addition of CT scans. In total, 63% of cases were recorded as unclassifiable by at least one surgeon, and 46% by at least two in the Hutt et al. Study.^9^ The Letournel and Judet classification system could be fail with acetabulum fractures which do not match the fracture lines described by Letournel and Judet, especially when quadrilateral surface fracture is included.^9^ Although the quadrilateral surface is an important anatomic structure and essential in the surgical reduction of fractures of the acetabulum, it is not a part of the systems developed for classifying these fractures.^11,12^ In the current study the overall Kappa value was found to be poor at 0.287 in comparison with the Hutt et al. study, in which 4 surgeons worked together, whereas the current study included 17 surgeons from 10 different centers and so it can be considered that their practices may be different. And as the Fleiss kappa methods when the observer number increased the kappa value decraese, kappa coefficient is highly affected by the number of observers.^10^ Herman et al. described a novel classification system as the vectors of trauma with 6 fracture patterns and according to their study, 20% of fractures could not be classified according to the Letournel and Judet classification system.^13^ The novel classification described by Herman et al. requires studies of interobserver agreement, additional data from 3D CT, and assessments of the overall effect on clinical outcomes. Ohashi et al. reported Kappa values of 0.42 for interobserver agreement of the classification of 101 acetabular fractures when only radiographs were viewed and 0.70 when only multidetector CT images were viewed.^14^ It was concluded in that study that plain radiographs are not necessary and CT scans are sufficient for classification. However, plain radiographs can detect a fracture line more frequently using AP pelvis radiographs, so patients can avoid unnecessary CT imaging with radiation exposure. In a study by Ohashi et al, there were only two radiologist observers, and it was stated that with a higher number of observers, agreement could change. Consistent with the findings of the current study, Visutipol et al. found that the addition of a 3D CT scan did not improve the inter- or intraobserver reliability of the Letournel classification with Kappa values reported of 0.42 for plain radiographs and 0.44 for 3D CT evaluation.^15^ But in the current study despite the non significant kappa increasing for CT images the percentage of agreement increse more. In the patient eight series the senior author's diagnosis was posterior column and the 70 % was posterior wall according to x rays, 58.8 % was posterior column according to 2D-CT scans and 76.5 % was posterior column according to 3D-CT scans.

Prevezas et al. used the iliopectineal and ilioischial lines to group fractures according to integrity and to then classify them according to the Letournel and Judet classification, yet they failed to demonstrate any significant improvement in concordance.^16^ Petrisor et al. conducted a comparative study between orthopaedic surgeons in training and those who had already graduated. Interobserver concordance using the Letournel and Judet classification was found to increase in direct relation to the surgeon's experience, regardless of the addition of oblique views.^17^ However, that study used four elementary fracture types and roof fracture and tear drop disruption, so the agreement would be expected to be higher and anterior wall fracture agreement poorer among all trained observers. In the current study, the maximum agreement was found between surgeons who performed 10-20 acetabular fracture operations per year.

Boudissa et al. studied semi-automatic bone-fragment segmentation through orthopaedic residents and found that agreement improved with semi-automatic bone-fragment segmentation but in the study, only fractures which could be classified were used.^18^ Riouallon et al. developed an application based on the Letournel and Judet classification. In that method, 8 radiographic landmarks were systematically examined for fracture lines, including 3 anterior landmarks (iliac wing, linea arcuata, and anterior wall of the acetabulum), 3 "no man's land" landmarks (roof of the acetabulum, quadrilateral surface, and obturator ring), and 2 posterior landmarks (posterior border of the iliac bone and posterior wall of the acetabulum). According to the study results, using the application improved reliability among 14 observers with different degrees of experience. However, the study was monocentric and there could have been selection bias in terms of the examiners.^19^ Clarke et al, the overall interobserver agreement for the Letournel and Judet classification was found to be moderate with a Kappa value of 0.52 in 4 four trauma centres and the highest Kappa value of 0.60 in the 3D CT set.^20^ In that study, a single set of images was evaluated but in the current study all 2D CT and 3D CT images were presented in video format and therefore, whole sets of images were given to the observers, who comprised 17 trauma surgeons from 10 different trauma centres. The plain radiographs, 2D CT and 3D CT videos were given in the same set and the participants were asked not to change the previous answers following 2D CT and 3D CT images. Thus the additional effect on interobserver reliability could be studied.

In conclusion, the results of this study revealed that the Letournel and Judet classification is not sufficient for the classification of acetabular fractures because of unclassified fractures and the complex algorithm. Even among experienced surgeons, interobserver raliability was found to be poor. Therefore, a clearer classification system is required for acetabular fractures
